# Cost-effectiveness of [¹¹C]Choline PET/CT as first-line imaging in primary hyperparathyroidism

**DOI:** 10.1007/s00259-025-07746-6

**Published:** 2026-02-09

**Authors:** H. M. Schouw, J Melis, J. W. Lutterop, H. H. Boersma, M. E. Noltes, C. S. van der Hilst, M. I. Bonnema, A. P. A. Appelman, W. T. Zandee, S. Kruijff, K. M. Vermeulen, A. H. Brouwers

**Affiliations:** 1 Department of Nuclear Medicine and Molecular Imaging, University of Groningen, University Medical Centre Groningen, Groningen, The Netherlands; 2 Department of Surgery, University of Groningen, University Medical Centre Groningen, Groningen, The Netherlands; 3 Department of Molecular Medicine and Surgery, Karolinska Institute, Stockholm, Sweden; 4 Department of Clinical Pharmacy and Pharmacology, University of Groningen, University Medical Centre of Groningen, Groningen, The Netherlands; 5 Department of Strategic Analytics, Finance and Control, University of Groningen, University Medical Centre of Groningen, Groningen, The Netherlands; 6 Department of Radiology, Groningen, University of Groningen, University Medical Centre Groningen, Groningen, The Netherlands; 7 Medical Centre Groningen, Department of Endocrinology, University of Groningen, University Medical Centre Groningen, Groningen, The Netherlands; 8 Department of Epidemiology, University of Groningen, University Medical Centre Groningen, Groningen, The Netherlands

**Keywords:** Primary hyperparathyroidism, Cost-utility, [^99m^Tc]Tc-methoxy isobutyl isonitrile SPECT/CT, [¹¹C]choline PET/CT

## Abstract

**Purpose:**

We performed a cost-utility analysis, using prospectively gathered trial data, comparing two imaging strategies for localizing parathyroid adenomas in primary hyperparathyroidism (pHPT) to determine the most cost-effective approach. Additionally, we provide customizable open-source R-script enabling other centres to identify their optimal imaging strategy based on local diagnostic accuracy and cost data.

**Methods:**

An evaluation of the diagnostic accuracy was performed for five imaging modalities: first-line cervical ultrasound (cUS) and [^99m^Tc]Tc-methoxy isobutyl isonitrile-single-photon-emission-computed- tomography/computed-tomography (MIBI SPECT/CT), and second-line [¹¹C]choline positron-emitting-tomography/CT (PET/CT), [¹¹C]methionine PET/CT, and 4 dimensional (4D)-CT. A decision-tree-model, constructed in R-studio, compared two diagnostic pathways: (1) The comparator pathway: a stepwise approach starting with cUS and MIBI SPECT/CT escalating to one of three second-line imaging modalities if needed, and (2) use of only one second-line imaging. Costs and quality-adjusted life years (QALYs) were evaluated across pathways, and cost-utility ratios (€/QALY) were calculated for a centre specific perspective with a 24-year time horizon based on life expectancy. In addition, to test the joint parameter uncertainty of the model, a probabilistic Monte-Carlo analysis was performed. One- and two-way sensitivity analyses were conducted to assess model robustness.

**Results:**

[¹¹C]choline PET/CT had a total costs of €10,394 and a QALY gain of 16.66. In contrast, the current standard, cUS + MIBI SPECT/CT with, when necessary, second-line imaging, costs €10,907 and yields 16.63 QALYs. The incremental cost-utility ratio (ICUR) for [¹¹C]choline PET/CT was -€18,846/QALY, indicating dominance with lower cost and greater effectiveness. Sensitivity analyses showed that cost-effectiveness was most sensitive to variations in costs of [¹¹C]choline PET/CT.

**Conclusion:**

This centre-specific model supports first-line [¹¹C]choline PET/CT as a cost-effective first-line strategy for localization of parathyroid adenomas, depending on [¹¹C]choline PET/CT costs. Additionally, the provided cost-utility model, enables other centres to determine their optimal imaging strategy.

**Supplementary Information:**

The online version contains supplementary material available at 10.1007/s00259-025-07746-6.

## Introduction

Primary hyperparathyroidism (pHPT) is caused by one or more hyperfunctioning parathyroid glands. It typically occurs in post-menopausal women and has a prevalence of 100–200 cases per 100.000 individuals [1, 2].

The only curative treatment for pHPT is surgical parathyroidectomy of the gland(s) [3–5]. When surgical intervention is necessary, a minimally invasive parathyroidectomy (MIP) is the preferred method. Due to the focussed approach a MIP typically results in a short recovery time, little postoperative pain, reduced scarring, and lower risk of complications such as recurrent laryngeal nerve damage or hypoparathyroidism [5, 6]. To perform a successful focussed MIP, it is important to localize the parathyroid adenoma(s) pre-operatively, for which several imaging modalities are available. Examples are cervical ultrasound (cUS), ^99m^Tc-sestamibi-single-photon-emission-computed tomography-computed-tomography (MIBI SPECT/CT), four-dimensional (4D-)CT, [^11^C]choline positron-emitting-tomography/computed-tomography ([^11^C]choline PET/CT) and [^11^C]methionine PET/CT. These imaging techniques differ in their specificity and sensitivity for detecting parathyroid adenomas [7–12]. Current guidelines recommend cUS combined with MIBI SPECT/CT as first-line imaging, due to its widespread availability and effectiveness [5, 13, 14].

In recent decades, the use of advanced imaging modalities such as [¹¹C]methionine PET/CT, [¹¹C] or [¹⁸F]choline PET/CT, and 4D-CT has increased, either as first-line localization or as second-line options when cUS and MIBI SPECT/CT yield inconclusive results [11, 12, 15]. A previous prospective study in our centre revealed that [^11^C]choline PET/CT was the most sensitive (85%) second-line modality of the aforementioned methods. However, economic considerations also play a role in determining the optimal imaging modality or strategy. Thus far, we did not investigate which of these imaging modalities is the most cost-effective in our specific hospital setting, and whether they should be implemented as first- or second-line options. Several studies have evaluated the cost-utility of imaging strategies for primary hyperparathyroidism (pHPT), using cost and sensitivity data from literature. However, the findings have been inconsistent. Some studies demonstrate quality-adjusted life year (QALY) gains with first-line [^18^F]fluorocholine PET/CT, while others report no significant benefit [13, 16–18].

The primary aim of this study is to identify the most cost-effective imaging strategy for pre-operative localization of parathyroid adenomas by evaluating the cost and performance quality, based on site specific diagnostic accuracy and healthcare costs data of several imaging modalities: cUS, MIBI SPECT/CT, [^11^C]methionine PET/CT, [ ^11^C]choline PET/CT, and 4D-CT). By incorporating site-specific prospective data on imaging performance, we aimed to ensure that the model accurately reflects local performance. As a secondary aim this study intends to provide a customizable model that helps other centres to cost-effectively tailor their pHPT imaging strategy to local circumstances.

## Methods

### Study population

The study population consisted of pHPT patients who underwent imaging for the localization of one or several parathyroid adenoma(s) with cUS, MIBI SPECT/CT, [^11^C]methionine PET/CT, [^11^C]choline PET/CT, and/or 4D-CT between the January 2019 and October 2022 in the University Medical Centre Groningen (UMCG). The study population consisted of two subgroups.

1. **The PARROT cohort**

This group consists of patients that were prospectively enrolled in the PARROT study during the study period, who underwent second-line imaging with [¹¹C]methionine PET/CT, [¹¹C]choline PET/CT, and 4D-CT after inconclusive or negative first-line imaging with cUS and MIBI SPECT/CT. This study was previously approved by the local ethics committee (METc UMCG, research registry number 2018/330) and clinical results have already been published [19]. PARROT patients who received first-line imaging, and/or treatment outside of the UMCG were excluded from the cost analysis.

2. **The non-PARROT cohort**

This group comprised patients that received imaging (cUS and/or MIBI SPECT/CT) in the UMCG during the same study period as the PARROT study but were not included in the PARROT study. The main reason for non-inclusion in the PARROT cohort was concordant positive first-line imaging.

Data collection for this retrospective cohort, as well as utilization of the PARROT data for this study, was locally approved by the Central Ethics Review Board (CTc UMCG, research registry number 11168) and was conducted in accordance with the declaration of Helsinki (October 2024).

Written informed consent was obtained from all patients included in both groups. Patients were excluded if they I] were known to have a germline mutation predisposing for multiple gland disease, II] if an alternative diagnosis (e.g. parathyroid carcinoma) was known before surgery, if they had III] a previous negative neck exploration for pHPT, IV] persistent pHPT after previous negative neck exploration, V] eGFR lower than 30 ml/min. VI] allergic to iodinated contrast agents (Points V] and VI] apply to the PARROT cohort only).

### Local performance analysis and model parameters

The probabilities associated with imaging outcomes were derived from the patient population at our hospital to tailor the model specifically to the diagnostic context of our institution. The combined sensitivity of cUS and MIBI SPECT/CT was assessed by evaluating how often the interpretation of these imaging modalities was classified as positive for localization during the weekly multidisciplinary team meeting at the UMCG. Subsequently, it was determined which percentage of these patients had a successful MIP or required conversion to a bilateral neck exploration (BNE). Since [^11^C]methionine PET/CT, [^11^C]choline PET/CT and 4D-CT were not utilized as first-line imaging in the UMCG during the study period, second-line performance data as previously published in the PARROT-study were used for the decision-tree-model [19].

Given the small size of the patient cohort used to estimate imaging probabilities and the low incidence of complications within this cohort, complications and long-term outcomes were considered more accurate when based on population-level data. Thus, probabilities for complications, treatment success, and life expectancy were derived from previously published literature.

### Costs analysis

A hospital perspective was adopted, in which all pHPT imaging and pHPT related hospital health care use was measured in units (numbers of visits, procedures etc.) and valued in monetary terms according to the hospital’s cost price in the year 2022. All treatment and imaging-related healthcare costs were collected for each individual patient from the retrospective cohort and the PARROT-cohort, beginning from the initial imaging procedure, and extending to their final surgical outpatient clinic appointment (Table 1). These cost data were obtained from our institution’s financial department to ensure accuracy and consistency in capturing all local prices. Costs made outside the hospital, such as visits to general practitioners or costs labelled to other indications, were not included. The costs were categorized in outpatient clinic visits, hospital admissions, surgery, pHPT related imaging, non-pHPT diagnostics (e.g., EKG’s) and laboratory tests. Surgery costs include all costs of the operating room, including consumables, anaesthetics and additional interventions such as ioPTH. Costs associated with long-term complications of persistent disease, such as vertebral fracture surgery and lithotripsy, were obtained from the Dutch National Health Institute [20]. As these costs could not be derived from the study population, they were adjusted based on incidence rates reported in the literature. (Table 1).

### Health effects

Utilities related to each step and outcome in the diagnostic, and therapeutic pathway were gathered from literature and are measured on a scale in which 0 represents death and 1 perfect health (Table 1). Patients who are cured from the disease experience a better quality of life compared to patients with persistent or untreated disease and its related complications. With these utilities QALY’s were calculated by calculating the sum of utilities over the expected lifetime horizon of 24 years specific to pHPT [16, 21].

**Table 1:** List of model parameters including local imaging performance analysis, surgical probabilities, costs, and utilities, with values (ranges, confidence intervals, or standard deviations), and costs (€), with references as listed

Parameter	Values for probabilistic analysis	Reference
Probability concordant positive cUS + MIBI SPECT/CT	0.47^‡^	UMCG, Retrospective cohort
Probability positive [^11^C]methionine PET/CT	0.72^‡^	UMCG [19]
Probability positive [^11^C]choline PET/CT	0.88^‡^	UMCG [19]
Probability positive 4D-CT	0.47 ^‡^	UMCG [19]
Probability successful planned MIP after only cUS + MIBI SPECT/CT	0.91 (R: 0.40–1.00)	UMCG, Retrospective cohort
Sensitivity [^11^C]methionine PET/CT	0.67 (CI: 0.48–0.82)	UMCG [19]
Sensitivity [^11^C]choline PET/CT	0.85 (CI: 0.68–0.95)	UMCG [19]
Sensitivity 4D-CT	0.39 (CI: 0.23–0.58)	UMCG [19]
Probability receiving MIP after conclusive imaging	1.00	Multidisciplinary team meeting UMCG
Probability receiving BNE after negative/inconclusive imaging	1.00	Multidisciplinary team meeting UMCG
Probability MIP converting into BNE	0.08 ^‡^	[22]
Probability full curation after MIP	0.915 ^‡^	[22]
Probability full curation after negative or inconclusive imaging followed by BNE	0.506 ^‡^	[22]
Probability persistent hyperparathyroidism after BNE	0.494 ^‡^	[22]
Probability full curation after exploration due to ioPTH	0.962 ^‡^	[22]
Probability persistent hyperparathyroidism after exploration due to ioPTH	0.038 ^‡^	[22]
Probability complications after MIP	0.015 ^‡^	[23]
Probability complications after BNE	0.031 ^‡^	[23]
Probability suffering secondary disorders due to persistent hyperparathyroidism	0.246 ^‡^	[24]
Costs MIP	€4455,- ^‡^	UMCG
Costs BNE	€5224,- ^‡^	UMCG
Costs conversion MIP into BNE	€769,- ^‡^	UMCG
Costs cervical ultrasound	€139,- ^‡^	UMCG
Costs MIBI SPECT/CT	€844,- ^‡^	UMCG
Costs [^11^C]methionine PET/CT	€810,- ^‡^	UMCG
Costs [^11^C]choline PET/CT	€810,- ^‡^	UMCG
Costs 4D-CT	€225,- ^‡^	UMCG
Costs clinic^†^	€2605,- ^‡^	UMCG
Costs outpatient clinic^†^	€954,- ^‡^	UMCG
Costs non-pHPT related diagnostics^†^	€109,- ^‡^	UMCG
Costs clinical chemistry and haematology^†^	€280,- ^‡^	UMCG
Costs microbiology^†^	€44,- ^‡^	UMCG
Costs pathology^†^	€180,- ^‡^	UMCG
Costs other care activities^†^	€861^‡^	UMCG
Other costs (pre-surgical assessment)^†^	€98,- ^‡^	UMCG
Costs lithotripsy^*^	1242.27 * (31/37) = €1041,-^‡^	[20, 24]
Costs vertebral fracture surgery^*^	7193.85 * (13/37) = €2528,- ^‡^	[20, 24]
Utility after full curation	0.839 (StDev: 0.176)	[25]
Disutility from complications due to surgery	0.179 (StDev: 0.036)	[25]
Utility persistent hyperparathyroidism with no secondary disorders	0.839 (StDev 0.179)	[25]
Utility suffering secondary disorders due to persistent hyperparathyroidism	0.590 (StDev 0.118)	[26, 27]
Life expectancy after diagnosis	24 (R: 12–36)	[16, 21]

^†^ The total of the cost related to these components were considered the costs related to these components for every pHPT patient and were added to every branch of the decision tree.

^*^ Cost related to these components were calculated and corrected for the incidence within the persistent HPT group as reported in literature and were added to this group in the decision tree.

^‡^ A range of 20% was used.

- Distributions: All costs and disutilities follow a Gamma-distribution, all probabilities and utilities follow Beta-distributions.

Cervical ultrasound (cUS), [99mTc]Tc-methoxy isobutyl isonitrile single photon emission computed tomography computed tomography (MIBI SPECT/CT

Positron emitting tomography/computed tomography (PET/CT), Minimal invasive parathyroidectomy (MIP), Bilateral neck exploration (BNE), intra-operative parathyroid hormone (ioPTH), 4-dimensional computed tomography (4D-CT), Primary hyperparathyroidism (pHPT), University Medical Centre Groningen (UMCG). Confidence interval (CI), Standard Deviation (StDev), Range (R).

### Modelling

**Fig. 1:**
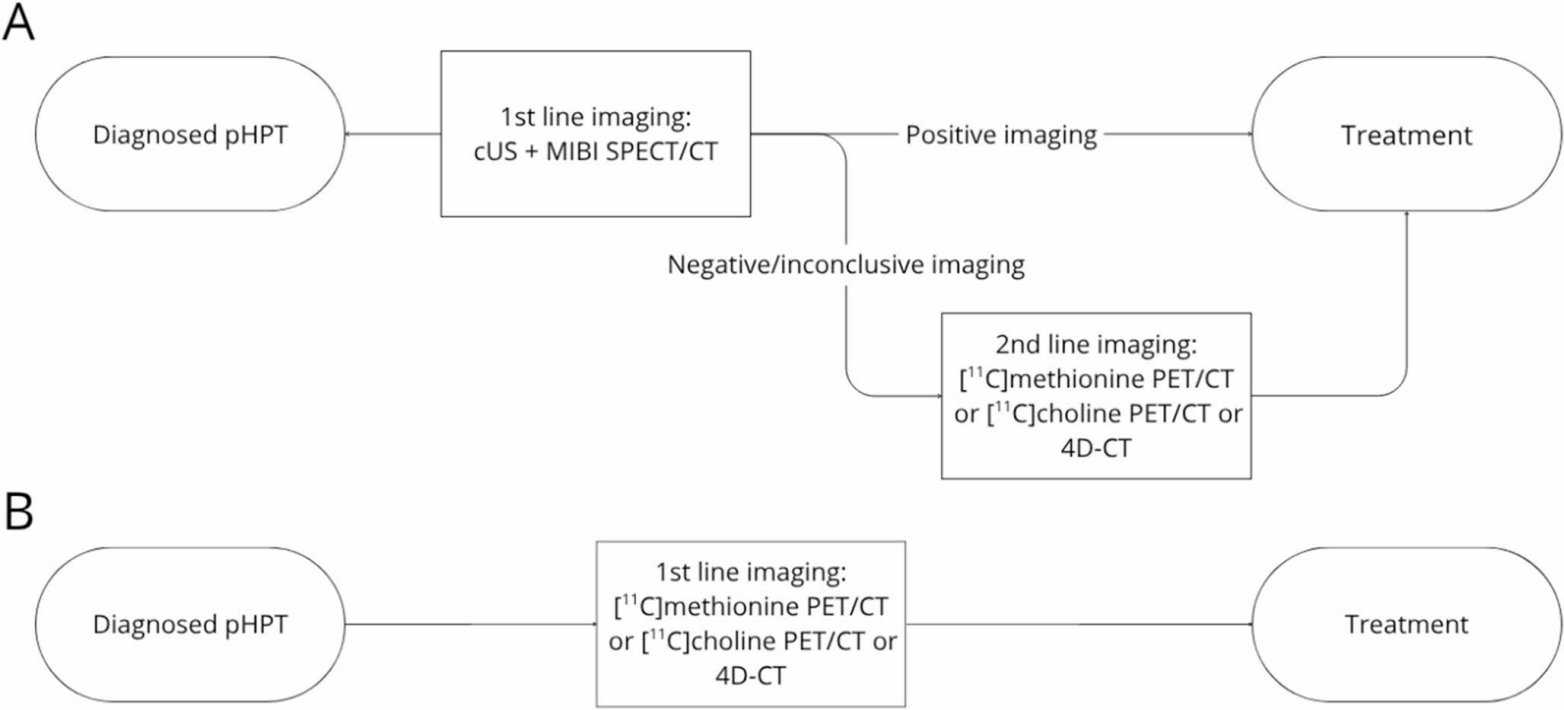
Imaging strategies to be simulated in model pictured in diagram. In A, the current stepwise practice is shown. In B a one-stop practice using only one of the second-line imaging techniques (3 options) as first-line imaging technique is shown. Abbreviations: [^99m^Tc]Tc-methoxy isobutyl isonitrile single photon emission computed tomography computed tomography (MIBI SPECT/CT), four-dimensional computed tomography (4D-CT), [^11^C]choline positron emitting tomography computed tomography ([^11^C]choline PET/CT) and [^11^C]methionine positron emitting tomography computed tomography ([^11^C]methionine PET/CT)

**Fig. 2:**
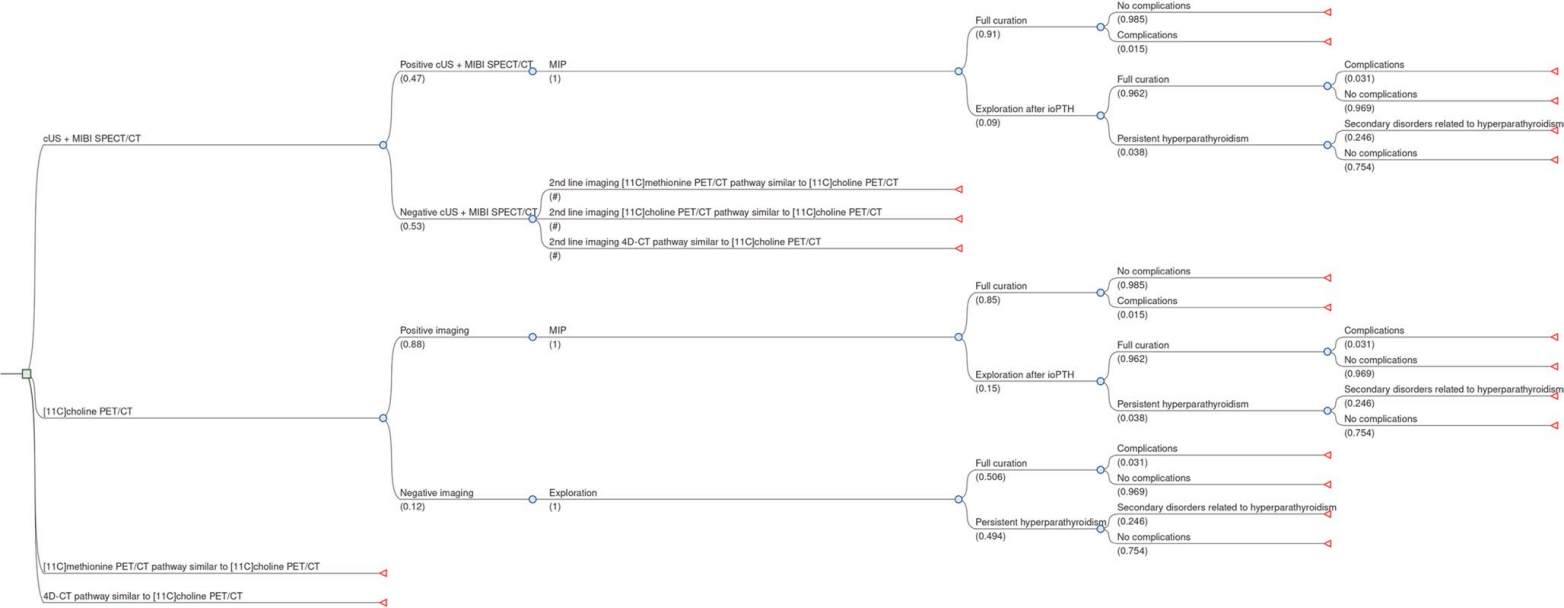
Decision tree model which depicts the imaging strategies and clinical scenarios that are possible for a patient with primary hyperparathyroidism (pHPT). A hypothetic patient follows the branches from left to right. As they move along the tree, medical costs accumulate according to the diagnostic and treatment pathways followed. Probabilities for the first node of [^11^C]methionine PET/CT and 4D-CT are listed in Table 1. Subsequent nodes in these strategies are identical to the corresponding nodes in the [^11^C]choline PET/CT pathway. A full version of the decision tree can be found in **Online Resource 2**. Of note, scenarios with repeated imaging and follow-up were not modelled. Abbreviations: [^99m^Tc]Tc-methoxy isobutyl isonitrile single photon emission computed tomography computed tomography (MIBI SPECT/CT), four-dimensional computed tomography (4D-CT), [^11^C]choline positron emitting tomography computed tomography [^[11^C]choline PET/CT) and [^11^C]methionine positron emitting tomography computed tomography ([^11^C]methionine PET/CT), intra-operative parathyroid hormone assay (ioPTH)

A decision tree was designed based on the clinical diagnostic and imaging pathway regarding pHPT at the UMCG and data from Table 1. To evaluate the cost-effectiveness of current imaging modalities (cUS, MIBI SPECT/CT, [¹¹C]methionine PET/CT, [¹¹C]choline PET/CT, and 4D-CT), several imaging strategies were simulated. In the first strategy, the comparator pathway is modelled: if first-line imaging (cUS and MIBI SPECT/CT) is negative or inconclusive, one of the second-line imaging modalities is employed, followed by the corresponding surgical approach (Fig. 1A). Three other imaging strategies are modelled: cUS and MIBI SPECT/CT are not used, and a ‘one-stop strategy’ is applied. Either [^11^C]methionine PET/CT, [^11^C]choline PET/CT or 4D-CT are used as first-line imaging modalities followed by the corresponding surgical treatment pathway **(**Fig. 1B**).** In the UMCG, intra-operative PTH (ioPTH) is used to assist in decision-making during surgery regarding the success of the MIP, and this is incorporated in the decision tree. Following the development of the decision tree (Fig. 2), it was implemented in RStudio (version 2025.05.0) using R (version 4.4.0) to conduct further analyses (**Online Resource 1**). Of note, scenarios with repeated reimaging and follow-up were not modelled. A hypothetical cohort of patients with pHPT was randomized across each arm of the decision tree. A base-case scenario was used to estimate the cost per QALY gained. The incremental cost-utility ratio (ICUR) was calculated relative to the current standard of care (cUS + MIBI SPECT/CT) over an expected lifetime horizon of 24 years according to the following formula: ICUR_modalityX_ = (Cost_modalityX_ – Cost_cUS+MIBI SPECT/CT_)/(QALY_modalityX_ – QALY_cUS+MIBI SPECT/CT_). The ICUR, expressed in €/QALY gained, was then compared to a willingness-to-pay threshold of €20,000, which is adjusted for disease burden in the Netherlands [28]. Discounting of 3.0% and 1.5% for costs and utilities, respectively, was applied, for all costs-utility gains after the first year following surgery [29].

### Threshold analysis

The model was subjected to a one-and two-way sensitivity analysis to test its robustness and to test its applicability to other hospitals scenario’s where different costs or performance of imaging modalities might apply. The one-way analysis assesses the effect of costs, performance and utility on the ICUR, whereas the two-way analysis will consider the price and sensitivity at the same time giving information at which threshold one modality dominates the other. For these analyses various model parameters were adjusted within their confidence intervals, as reported in the literature or as calculated. When no confidence interval or literature reference was available, a ± 20% range was applied [29]. To test the joint parameter uncertainty of the model a probabilistic Monte-Carlo analysis was performed. A total of 100.000 simulations were performed with parameter ranges and distributions as reported in Table 1. The results were plotted in a cost-effectiveness acceptability curve (CEAC) which depicts the probability of a modality being cost-effective compared to the current clinical standard (MIBI SPECT/CT) as a function of the WTP.

## Results

### Local performance analysis, model parameters and costs

**Fig. 3:**
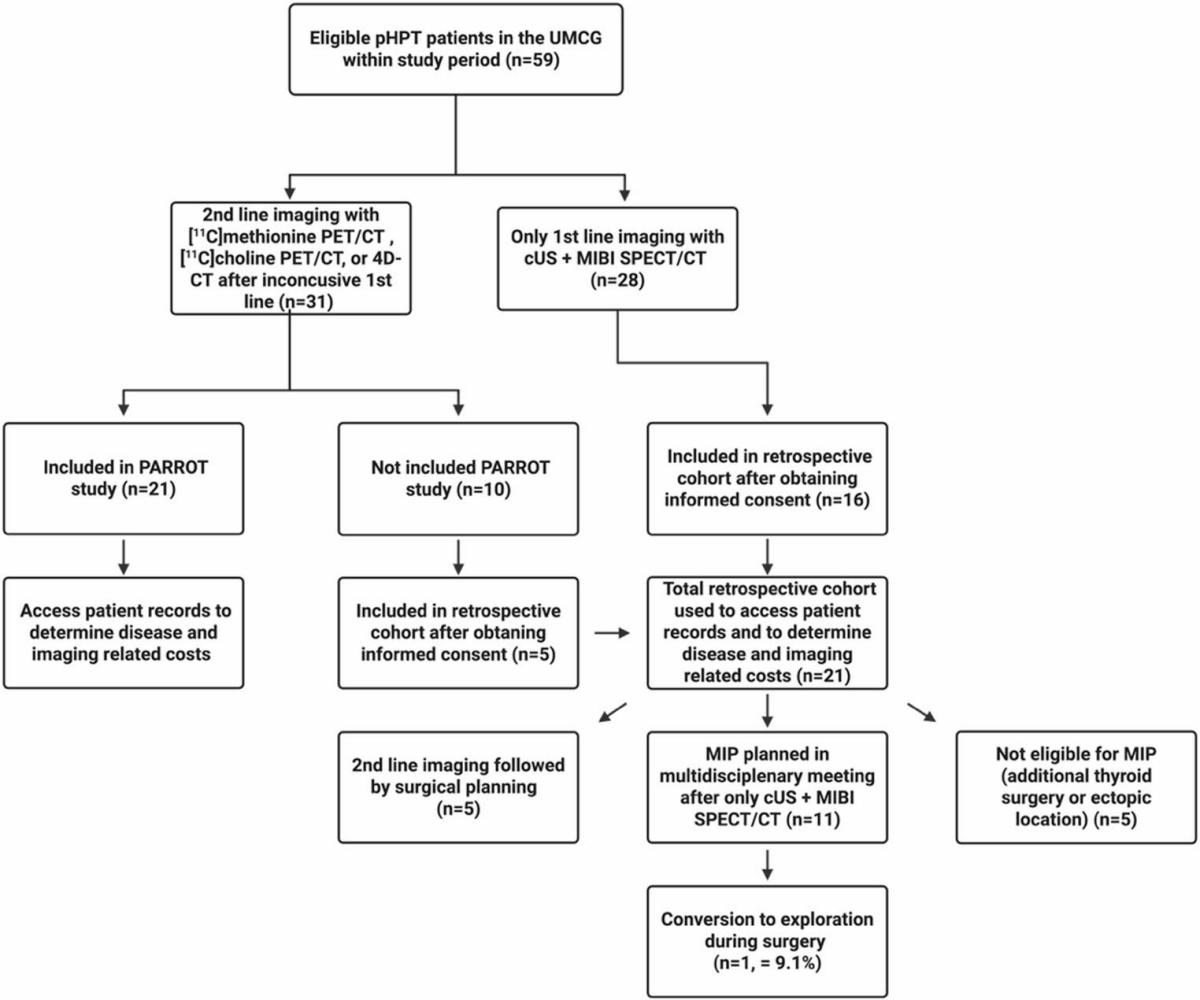
Flowchart describing how all eligible primary hyperparathyroidism (pHPT) patients in the UMCG were included in the previous PARROT study and the retrospective cohort. Five out of the 21 patients included in the retrospective cohort received 2nd line imaging. Five of the 16 remaining patients were considered not eligible for a minimal invasive parathyroidectomy (MIP) due to simultaneous thyroid surgery or other anatomical challenges. One of the 11 planned MIP’s was converted to an exploration. Abbreviations: [ ^99m^Tc]Tc-methoxy isobutyl isonitrile single photon emission computed tomography computed tomography (MIBI SPECT/CT), four-dimensional computed tomography (4D-CT), [^11^C]choline positron emitting tomography computed tomography( [^[11^C]choline PET/CT) and [^11^C]methionine positron emitting tomography computed tomography ([^11^C]methionine PET/CT)

During the study period, a total of 59 patients diagnosed with primary hyperparathyroidism (pHPT) underwent imaging at the UMCG only. A flowchart showing the process of patient inclusion is depicted in Fig. 3. After obtaining informed consent, 21 of 38 eligible patients were included in the retrospective cohort. This cohort was used to evaluate imaging performance based on surgical outcomes described in the patient records and to determine patient-specific costs. Of the 21 patients included in the retrospective cohort, five had received additional imaging after cUS + MIBI SPECT/CT. Focusing specifically on the 16 patients from this group who had only cUS + MIBI SPECT/CT: 11 patients were considered eligible for a minimally invasive parathyroidectomy (MIP) after a multidisciplinary team meeting, of which one patient required conversion to a BNE (9.1%).

Probabilities related to the local performance of the imaging modalities (UMCG), probabilities related to the surgical trajectory (literature) and related costs are depicted in Table 1.

Surgical costs of a MIP vs. a BNE were determined based on the documented surgical costs of MIP patients vs. BNE patients.

### The decision tree model

Patients progress through the decision tree by following branches determined by the probabilities assigned to each decision or chance node. As they move along the tree, medical costs accumulate based on the diagnostic and treatment pathways followed. At each terminal (end) node, a QALY value is assigned to reflect the patient’s final health outcome.

**Fig. 4:**
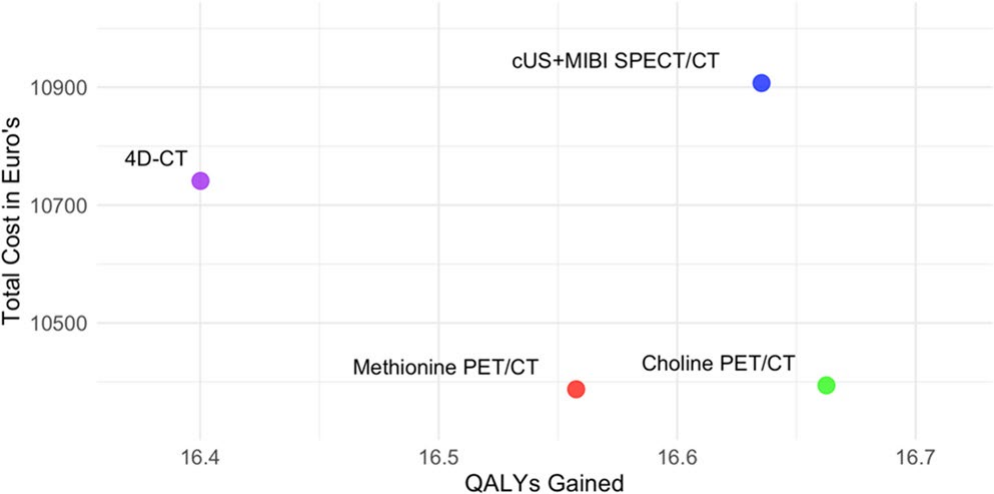
Relative cost-utility ratios of the included imaging modalities for diagnosis of primary hyperparathyroidism (pHPT). Abbreviations: QALY: quality adjusted life year; cUS+MIBI SPECT/CT: cervical ultrasound + single photon emission computed tomography/computed tomography using [⁹⁹ᵐTc]Tc-methoxy isobutyl isonitrile; 4D-CT: four-dimensional computed tomography; Choline PET/CT: [¹¹C]choline positron emission tomography/computed tomography; Methionine PET/CT: [¹¹C]methionine positron emission tomography/computed tomography

The base case analysis, using the decision tree, resulted in a relative cost-utility ratio for each imaging modality (Fig. 4). This analysis revealed that the one-stop [^11^C]choline PET/CT costs €10,394 and gained 16.66 QALYs. The one-stop [^11^C]methionine PET/CT strategy had a total cost of €10,388 and gained 16.55 QALYs. The strategy with 4D-CT as first-line modality came with a total cost of €10,741 and gained 16.40 QALYs. The current clinical standard of cUS + MIBI SPECT/CT followed by possible second-line imaging resulted in a total cost of €10,907 and 16.64 QALYs gained.

**Fig. 5:**
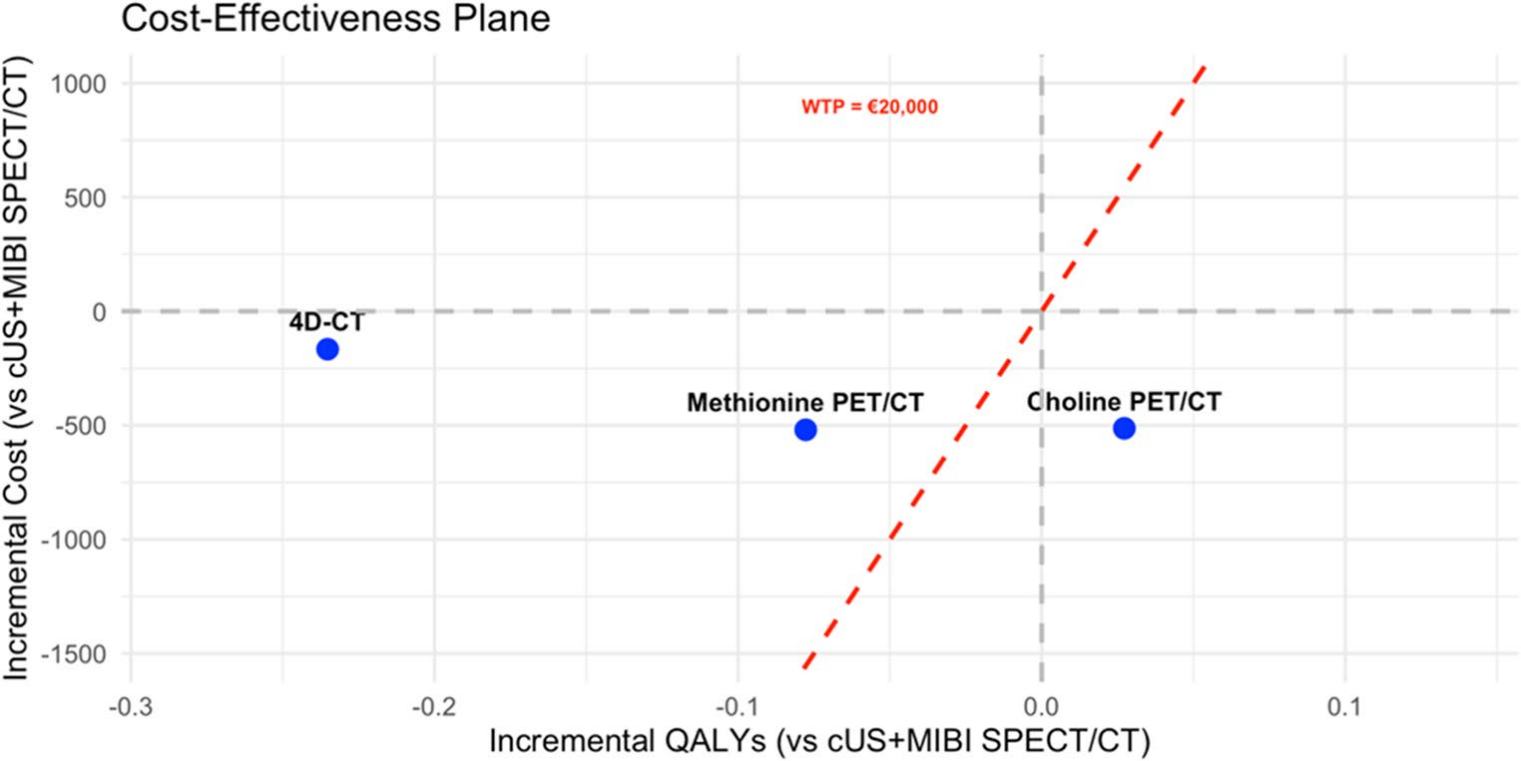
A cost-effectiveness plane displaying incremental costs and incremental quality-adjusted life years (QALYs) of [¹¹C]choline PET/CT, [¹¹C]methionine PET/CT, and 4D-CT compared to the current clinical standard of cervical ultrasound (cUS) + [⁹⁹ᵐTc]Tc-methoxy isobutyl isonitrile-single-photon-emission-computed-tomography/computed tomography (MIBI SPECT/CT). Results are evaluated against a willingness-to-pay threshold (WTP) of €20,000 [23]

Since cUS+MIBI/SPECT followed by second-line imaging, when necessary, was the current clinical standard, we assessed the relative and incremental cost-effectiveness of using second-line imaging modalities ([¹¹C]methionine PET/CT, [¹¹C]choline PET/CT, and 4D CT) as first-line options compared to the standard approach (cUS + MIBI SPECT/CT) (Fig. 5).

Compared to cUS + MIBI SPECT/CT, [¹¹C]choline PET/CT is providing higher QALYs at a lower cost while remaining below the willingness-to-pay (WTP) threshold of €20,000. In contrast, 4D-CT and [¹¹C]methionine-PET/CT are cheaper or similar in incremental costs but result in fewer QALYs and exceed the WTP threshold. For further analysis, we focused on comparing [¹¹C]choline PET/CT with cUS + MIBI SPECT/CT. One-way threshold analysis for 4D-CT and [¹¹C]methionine-PET/CT can be found in **Online Resource 3**.

The ICUR of [^11^C]choline PET/CT was calculated by using the following formula: ICUR_Choline_ = (Cost_Choline_ – Cost_cUS+MIBI SPECT/CT_)/(QALY_Choline_ – QALY_cUS+MIBI SPECT/CT_). This calculation resulted in finding an ICUR of -€18,846/QALY, meaning for each QALY gained the [^11^C]choline one-stop imaging strategy saved €18,846 in comparison to the current cUS + MIBI SPECT/CT imaging strategy.

### Threshold Analysis

**Fig. 6:**
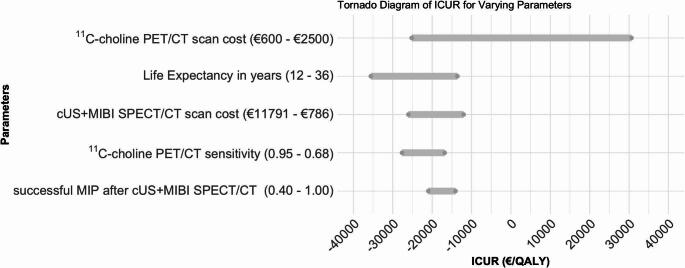
A tornado plot illustrating the impact of key parameters on the incremental cost-utility ratio (ICUR) of choline PET/CT compared to the standard approach of cervical ultrasound (cUS) combined with [⁹⁹ᵐTc]Tc-methoxy isobutyl isonitrile-single photon emission computed tomography/computed tomography (MIBI SPECT/CT). The modelled range for each parameter is indicated in brackets

 Figure 6 presents a tornado plot illustrating the results of the one-way sensitivity analysis on the ICUR of [¹¹C]choline PET/CT. The model was most sensitive to changes in [^11^C]choline PET/CT scan costs, while variations in the sensitivity of [¹¹C]choline PET/CT and the chance of a successful MIP after cUS + MIBI SPECT/CT had the least effect. Across plausible parameter ranges, derived from literature values (Table 1) or 20% ranges for cost estimates, the ICUR remains negative for all variables except the [¹¹C]choline PET/CT cost. This indicates that at a price of €1455,- [^11^C]choline PET/CT stops being the dominant strategy over cUS + MIBI SPECT/CT. Furthermore, at €2139,- the [^11^C]choline PET/CT costs exceed the WTP threshold of €20,000,-.

**Fig. 7:**
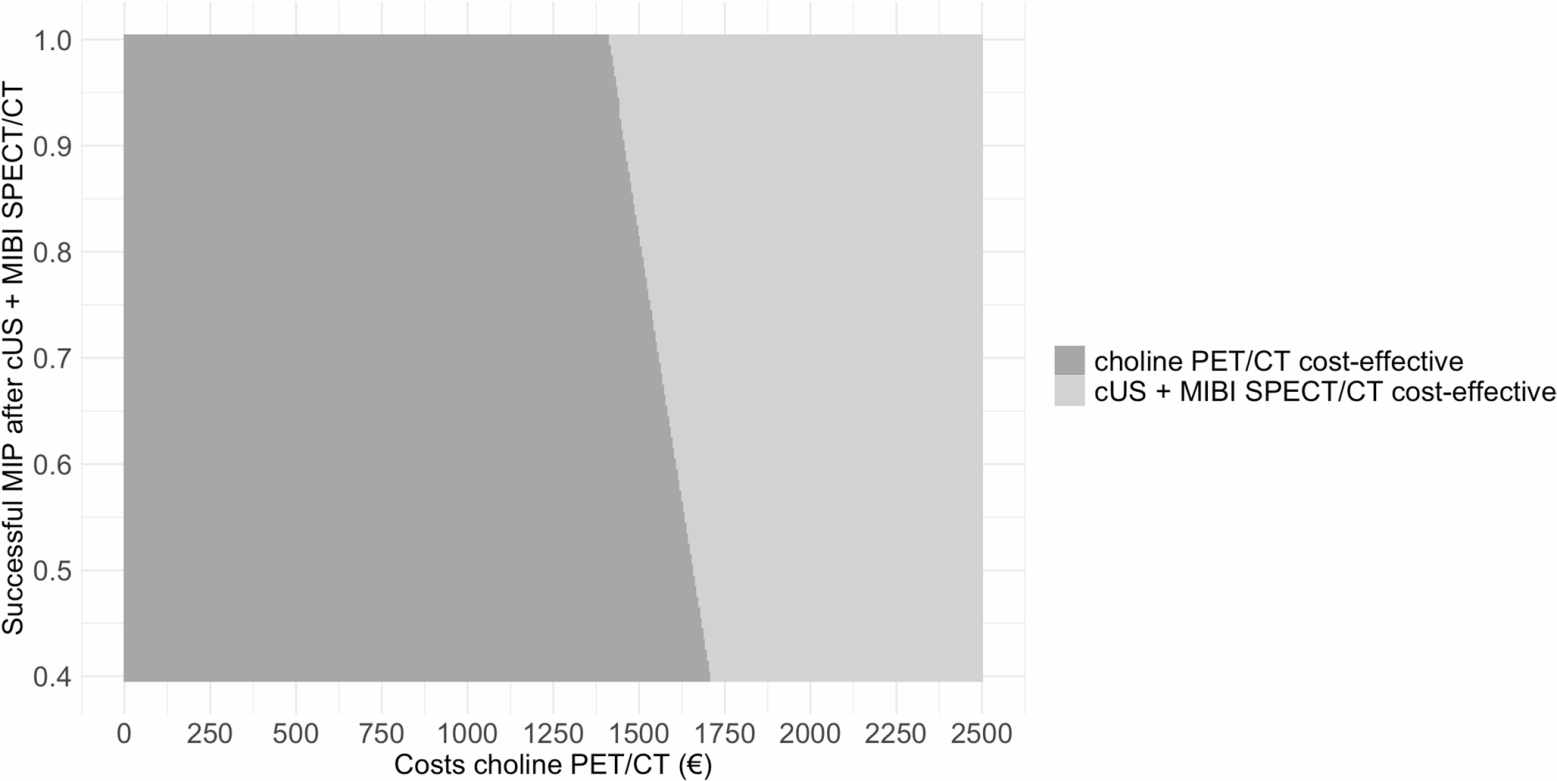
Two-way sensitivity analysis demonstrating how changes in the cost of [¹¹C]choline positron emitting tomography/computed tomography (PET/CT) and the sensitivity of combined cervical ultrasound (cUS) and[ ⁹⁹ᵐTc]Tc-methoxy isobutyl isonitrile single photon emission computed tomography/computed tomography (MIBI SPECT/CT) affect the dominance of each imaging strategy. The light grey area indicates where cUS+MIBI SPECT/CT becomes the dominant strategy over [¹¹C]choline PET/CT

 In the two-way sensitivity the price of [^11^C]choline PET/CT and the combined sensitivity of cUS + MIBI SPECT/CT were adjusted over a reasonable range to facilitate the application of our model to other medical centres (Fig. 7). cUS + MIBI SPECT/CT becomes the dominant strategy when [^11^C]choline PET/CT costs between approximately €1540,- and €1710,- across a cUS + MIBI SPECT/CT sensitivity range of 0.4 to 1.0

**Fig. 8:**
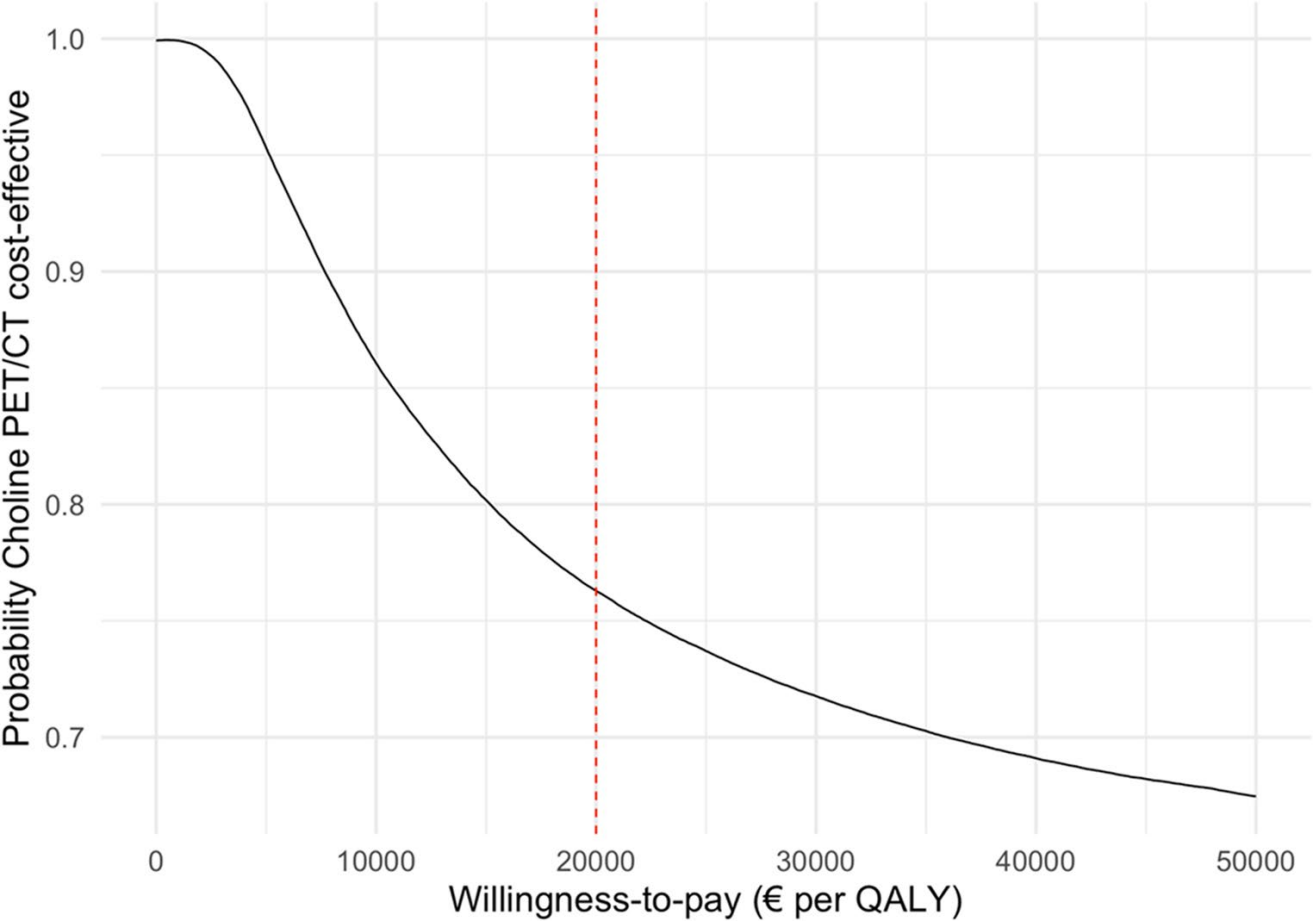
Cost effectiveness acceptability curve showing the probability that [¹¹C]choline positron emitting tomography/computed tomography (choline PET/CT) is cost-effective compared to the current clinical standard of [⁹⁹ᵐTc]Tc-methoxy isobutyl isonitrile single photon emission computed tomography/computed tomography (MIBI SPECT/CT) for a range of willingness-to-pay thresholds

 The cost-effectiveness acceptability curve resulting from the probabilistic sensitivity (Monte-Carlo) analysis, shown in Figure 8, illustrates the probability that choline PET/CT is cost-effective compared to MIBI SPECT/CT across a range of WTP thresholds. At a WTP threshold of €20,000 per additional unit of effect, the probability that choline PET/CT is cost-effective is 0.77.

##  Discussion

This study conducted a centre-specific cost-utility analysis of different imaging strategies for the preoperative localization of parathyroid adenomas using prospectively gathered trial data, diverging from the more common healthcare based perspective used in previous research, mainly relying on population registry data [16, 17]. In our centre-specific analysis, first-line use of [¹¹C]choline PET/CT was found to be cost-effective compared to the conventional imaging pathway involving initial cUS and MIBI SPECT/CT, followed by second-line imaging when first-line results were negative or inconclusive. However, this conclusion is sensitive to the cost of [¹¹C]choline PET/CT; at a threshold of €1,540 per scan, this strategy, depending on all other parameters, may lose its dominance over cUS + MIBI SPECT/CT. Nevertheless, the probabilistic Monte-Carlo analysis reveals that choline PET/CT has a 0,77 probability of being cost-effective at a WTP of €20.000. Since this conclusion is based on centre-specific costs and imaging performance, a model is provided (**Online Resource 1**) that can be adapted to evaluate cost-utility scenarios in different centres.

A previous study already showed that combining cUS and MIBI SPECT/CT improves diagnostic accuracy and reduces costs compared to using either alone [13]. Another study found that [¹⁸F]fluorocholine PET/CT may be a cost-effective option for primary hyperparathyroidism, with a more favourable ICUR than 4D-CT when used as a first-line modality, depending on its sensitivity and cost [16]. However, the findings were limited by the extrapolation of European cost data to the U.S. context [16]. A study by Van Mossel et al. compared first-line [¹⁸F]fluorocholine PET/CT to a stepwise strategy using cUS and MIBI SPECT/CT followed by [¹⁸F]fluorocholine PET/CT when results were negative or discordant [17]. The authors found that the simulated health outcomes and costs were comparable between both strategies, suggesting that they may be used interchangeably. Notably, their estimated incremental costs were substantially lower than those calculated in our study (€4,500 versus €10,000). This difference may be attributable to their use of a broad healthcare perspective, whereas our analysis adopted a local perspective and was partially based on prospectively collected data. Consistent with the findings of Van Mossel et al., we emphasize that real-world clinical practice plays a crucial role in shaping decisions regarding cost-effective imaging strategies [17]. Factors such as local performance of imaging modalities, resource capacity, reduction of hospital waste, waiting times and accepted radiation burden influence the scenarios in a cost-effectiveness analysis and therefore also warrant a local analysis, besides a healthcare-based analysis. For example, the centre where this study was conducted has the capability to produce [¹¹C]choline on-site, in contrast to many other clinics that use [¹⁸F]fluorocholine as a tracer for pHPT, which is often purchased and transported from other sites at higher costs. As a result, [¹⁸F]fluorocholine is often more expensive, with reported costs close to, or exceeding, €2,000, compared to the €810 reported for [^11^C]choline at the UMCG [16, 30]. The differences in logistic constraints related to [^11^C]choline and [¹⁸F]fluorocholine will affect the generalizability of our results. However, by providing the R code in **Online Resource 1**, other institutions might investigate which imaging strategy is cost effective in their local setting.

Additionally, we found that in our centre, following multidisciplinary discussion, only 47% (28/59) of the patients required no additional imaging after cUS + MIBI SPECT/CT. But when these patients were planned for a MIP without any additional imaging, this was successful in 91% (10/11) of patients operated in the UMCG. To enhance the generalizability of the results to other medical centres, the two-way sensitivity analysis varied the cost of [¹¹C]choline PET/CT up to €2,500 and the likelihood of a successful MIP in this group between 40% and 100%.

This study was subjected to a few limitations. Firstly, the centre-based model parameters related to scan performance were based on a relatively small sample size. Of all eligible patients, the data of *n* = 17 patients could not be used due to retrospectively not receiving informed consent, which may have introduced selection bias. Additionally, the effectiveness of [^11^C]methionine PET/CT, [^11^C]choline PET/CT, and 4D-CT was assessed in second-line settings, which may underestimate their performance in first-line populations, where sensitivity could exceed 90% [31]. To account for this, values above 90% were included in both the one-way and two-way sensitivity analyses. Furthermore, some costs included in the model were hard to determine. For example, the difference in surgical costs between a MIP and a BNE was only €769, based on the cost difference observed between these patient groups. This estimate primarily reflects standard reimbursement tariffs and treatment-related costs. However, factors such as extended operative time and potential delays in subsequent procedures, requiring increased staffing, may significantly raise costs, potentially leading to an underestimation of the true cost difference between MIP and BNE. Also, it is important to note that in real-world clinical practice, patients may undergo repeated imaging during prolonged follow-up, which was not incorporated in the present analysis. In addition, cUS might be also used parallel to PET or CT imaging possibly altering costs and outcomes of the pathways. These factors in total may influence both costs and outcomes. Additionally, given the small effect differences observed, the ICUR may be highly unstable and sensitive to minor variations in costs or QALYs. This underscores the need to jointly assess uncertainty to prevent potential misinterpretation of the cost-effectiveness results. This study used the combined application of cUS and MIBI SPECT/CT as first-line and compared this to a one-stop strategy where only [^11^C]methionine PET/CT, [^11^C]choline PET/CT or 4D-CT is applied. In many centres cUS will often be used side by side with either one of these modalities, for instance to rule out thyroid disease. If this is warranted depends on the local situation but could be deemed unnecessary in the case of no focal choline uptake in the thyroid, since choline PET/CT has a good negative prediction value for thyroid cancer, as has been previously investigated [32]. Alternatively, costs of pre-operative cUS could be added to the ‘one-stop’ branch of the decision tree. A significant impact on QALYs is not expected due to limited diagnostic impact.

For the decision-tree-model we used the combined sensitivity of cUS and MIBI SPECT/CT, defined by the number of patients undergoing parathyroid surgery without additional imaging and achieving successful MIP without conversion. This highlights the value of routine multidisciplinary meetings, where surgeons, endocrinologists, radiologists, and nuclear medicine physicians interpret scans in clinical context. In some cases, a positive result from either cUS or MIBI SPECT/CT alone was sufficient to proceed with MIP, often successfully.

In summary, this study is the first to evaluate the cost-effectiveness of imaging strategies using prospective data for pHPT from a local healthcare perspective. Our findings suggest that first-line use of [¹¹C]choline PET/CT is cost-effective in our centre and potentially in other institutions with similar infrastructure and imaging performance. For centres with varying diagnostic capabilities or workflows, the decision-analytic model in **Online Resource 1** offers open source R-script that can be used to identify the most cost-effective imaging strategy tailored to local practice. The decision tree can be easily adapted by adjusting branches, such as removing [¹¹C]choline or methionine PET/CT where unavailable or updating cost inputs to reflect local pricing and protocols. This model combined with previously published evidence on cost-effectiveness from a healthcare based perspective can support informed, centre-specific decision-making regarding the adoption of a one-stop-shop imaging approach for managing pHPT.

## Supplementary Information

Below is the link to the electronic supplementary material.

Supplementary Material 1

Supplementary Material 2

Supplementary Material 3

## Data Availability

The R model made in R-studio is provided as **Online Resource 1**. Data can be found in the Online Resources. Original datasets generated during and/or analysed during the study are available from the corresponding author on reasonable request.
