# Supplementary material for: Cost-effectiveness of [¹¹C]Choline PET/CT as first-line imaging in primary hyperparathyroidism
Source: Eur J Nucl Med Mol Imaging. 2026 Feb 9;53(6):4186–98. doi: 10.1007/s00259-025-07746-6 (PMC13121269; doi:10.1007/s00259-025-07746-6)
Supplement: Supplementary file 1 — Supplementary Material 1 [file 259_2025_7746_MOESM1_ESM.pdf]

Cost-Effectiveness of [^{11}C]Choline PET/CT as First-line Imaging in Primary Hyperparathyroidism

H.M. **Schouw,** PharmD, MD[1,2,3], J. **Melis,** MSc[1,4], J.W. **Lutterop,** MSc[2], H.H. **Boersma,** PharmD, PhD[1,4]**, M.E. Noltes,** MD, PhD[2], C.S. **van der Hilst,** PhD[5], M.I. **Bonnema,** MSc[5], A.P.A. **Appelman,** MD, PhD[6], W.T. **Zandee,** MD, PhD[7], S. **Kruijff,** MD, PhD[1,2,3], K.M. **Vermeulen,** PhD[8], A.H. **Brouwers,** MD, PhD[1]

1. University of Groningen, University Medical Centre Groningen, Department of Nuclear Medicine and Molecular Imaging, Groningen, The Netherlands
2. University of Groningen, University Medical Centre Groningen, Department of Surgery, Groningen, The Netherlands
3. Karolinska Institute, Department of Molecular Medicine and Surgery, Stockholm, Sweden
4. University of Groningen, University Medical Centre of Groningen, Department of Clinical Pharmacy and Pharmacology, Groningen, The Netherlands
5. University of Groningen, University Medical Centre of Groningen, Department of Strategic Analytics, Finance and Control, Groningen, The Netherlands
6. University of Groningen, University Medical Centre Groningen, Department of Radiology, Groningen, The Netherlands
7. University of Groningen, University Medical Centre Groningen, Department of Endocrinology, Groningen, The Netherlands
8. University of Groningen, University Medical Centre Groningen, Department of Epidemiology, Groningen, The Netherlands.

Corresponding author: H.M. Schouw, h.m.schouw@umcg.nl

Online Resource 2

Complete decision tree

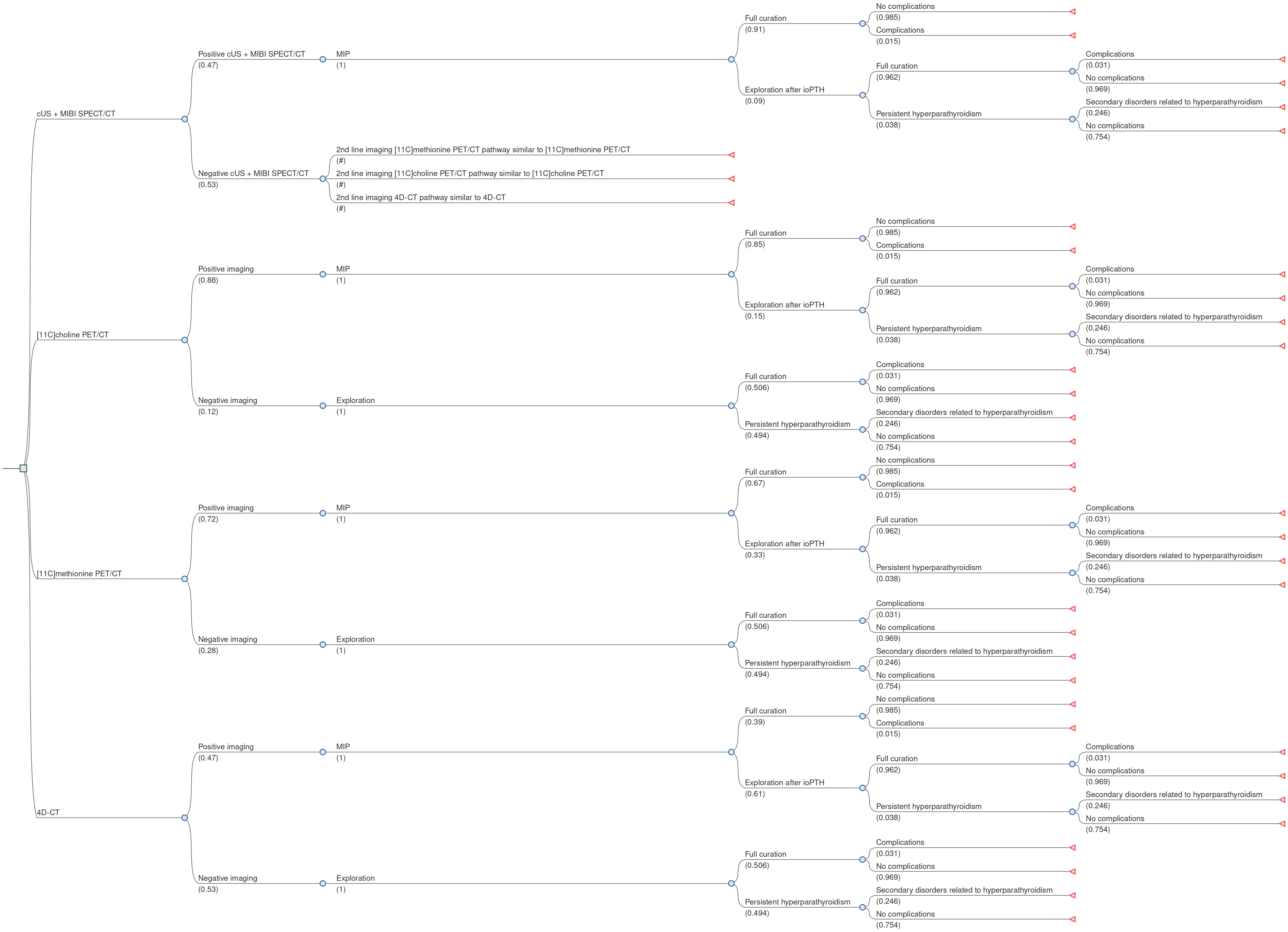
cUS + MIBI SPECT/CT
Positive cUS + MIBI SPECT/CT
(0.47)
MIP
(1)
Full curation
(0.91)
No complications
(0.985)
Complications
(0.015)
Exploration after ioPTH
(0.09)
Full curation
(0.962)
Complications
(0.031)
No complications
(0.969)
Persistent hyperparathyroidism
(0.038)
Secondary disorders related to hyperparathyroidism
(0.246)
No complications
(0.754)
Negative cUS + MIBI SPECT/CT
(0.53)
2nd line imaging [11C]methionine PET/CT pathway similar to [11C]methionine PET/CT
(#)
2nd line imaging [11C]choline PET/CT pathway similar to [11C]choline PET/CT
(#)
2nd line imaging 4D-CT pathway similar to 4D-CT
(#)
[11C]choline PET/CT
Positive imaging
(0.88)
MIP
(1)
Full curation
(0.85)
No complications
(0.985)
Complications
(0.015)
Exploration after ioPTH
(0.15)
Full curation
(0.962)
Complications
(0.031)
No complications
(0.969)
Persistent hyperparathyroidism
(0.038)
Secondary disorders related to hyperparathyroidism
(0.246)
No complications
(0.754)
Negative imaging
(0.12)
Exploration
(1)
Full curation
(0.506)
Complications
(0.031)
No complications
(0.969)
Persistent hyperparathyroidism
(0.494)
Secondary disorders related to hyperparathyroidism
(0.246)
No complications
(0.754)
[11C]methionine PET/CT
Positive imaging
(0.72)
MIP
(1)
Full curation
(0.67)
No complications
(0.985)
Complications
(0.015)
Exploration after ioPTH
(0.33)
Full curation
(0.962)
Complications
(0.031)
No complications
(0.969)
Persistent hyperparathyroidism
(0.038)
Secondary disorders related to hyperparathyroidism
(0.246)
No complications
(0.754)
Negative imaging
(0.28)
Exploration
(1)
Full curation
(0.506)
Complications
(0.031)
No complications
(0.969)
Persistent hyperparathyroidism
(0.494)
Secondary disorders related to hyperparathyroidism
(0.246)
No complications
(0.754)
4D-CT
Positive imaging
(0.47)
MIP
(1)
Full curation
(0.39)
No complications
(0.985)
Complications
(0.015)
Exploration after ioPTH
(0.61)
Full curation
(0.962)
Complications
(0.031)
No complications
(0.969)
Persistent hyperparathyroidism
(0.038)
Secondary disorders related to hyperparathyroidism
(0.246)
No complications
(0.754)
Negative imaging
(0.53)
Exploration
(1)
Full curation
(0.506)
Complications
(0.031)
No complications
(0.969)
Persistent hyperparathyroidism
(0.494)
Secondary disorders related to hyperparathyroidism
(0.246)
No complications
(0.754)
