# Supplementary material for: Cost-effectiveness of [¹¹C]Choline PET/CT as first-line imaging in primary hyperparathyroidism
Source: Eur J Nucl Med Mol Imaging. 2026 Feb 9;53(6):4186–98. doi: 10.1007/s00259-025-07746-6 (PMC13121269; doi:10.1007/s00259-025-07746-6)
Supplement: Supplementary file 2 — Supplementary Material 2 [file 259_2025_7746_MOESM2_ESM.pdf]

Cost-Effectiveness of [^{11}C]Choline PET/CT as First-line Imaging in Primary Hyperparathyroidism

H.M. **Schouw,** PharmD, MD[1,2,3], J. **Melis,** MSc[1,4], J.W. **Lutterop,** MSc[2], H.H. **Boersma,** PharmD, PhD[1,4]**, M.E. Noltes,** MD, PhD[2], C.S. **van der Hilst,** PhD[5], M.I. **Bonnema,** MSc[5], A.P.A. **Appelman,** MD, PhD[6], W.T. **Zandee,** MD, PhD[7], S. **Kruijff,** MD, PhD[1,2,3], K.M. **Vermeulen,** PhD[8], A.H. **Brouwers,** MD, PhD[1]

1. University of Groningen, University Medical Centre Groningen, Department of Nuclear Medicine and Molecular Imaging, Groningen, The Netherlands
2. University of Groningen, University Medical Centre Groningen, Department of Surgery, Groningen, The Netherlands
3. Karolinska Institute, Department of Molecular Medicine and Surgery, Stockholm, Sweden
4. University of Groningen, University Medical Centre of Groningen, Department of Clinical Pharmacy and Pharmacology, Groningen, The Netherlands
5. University of Groningen, University Medical Centre of Groningen, Department of Strategic Analytics, Finance and Control, Groningen, The Netherlands
6. University of Groningen, University Medical Centre Groningen, Department of Radiology, Groningen, The Netherlands
7. University of Groningen, University Medical Centre Groningen, Department of Endocrinology, Groningen, The Netherlands
8. University of Groningen, University Medical Centre Groningen, Department of Epidemiology, Groningen, The Netherlands.

Corresponding author: H.M. Schouw, h.m.schouw@umcg.nl

Online Resource 3

One-way analysis for 4D-CT and Methionine PET/CT

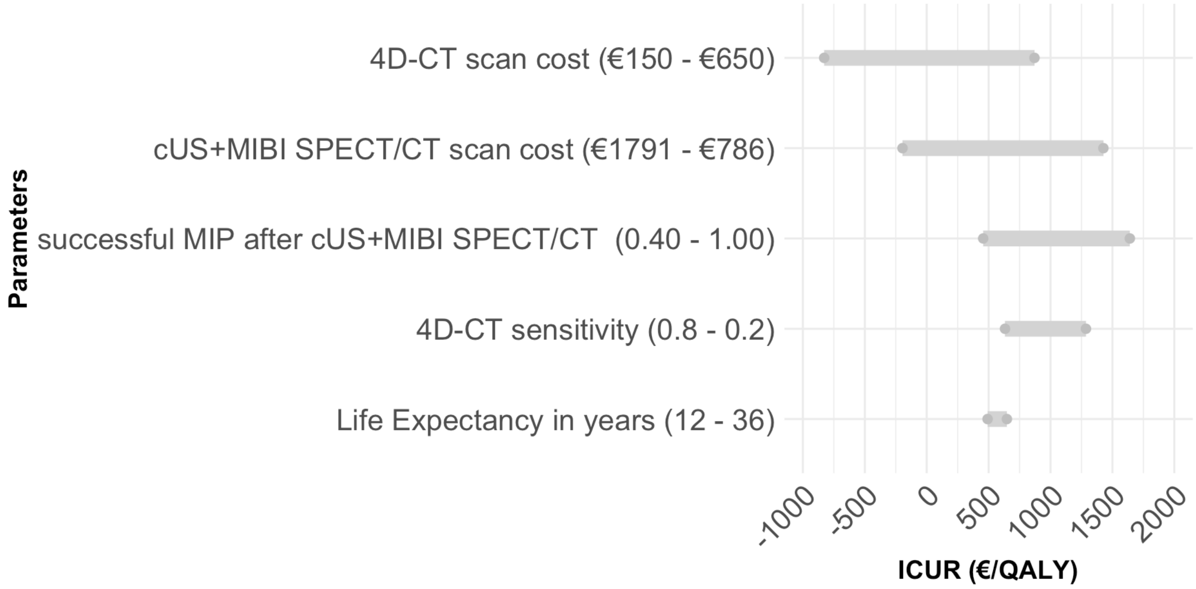

Figure 1: A tornado plot illustrating the impact of key parameters on the incremental cost-utility ratio (ICUR) of 4D-CT compared to the standard approach of cervical ultrasound (cUS) combined with [^{99m}Tc]Tc-methoxy isobutyl isonitrile-single photon emission computed tomography/computed tomography (MIBI SPECT/CT). The modelled range for each parameter is indicated in brackets.

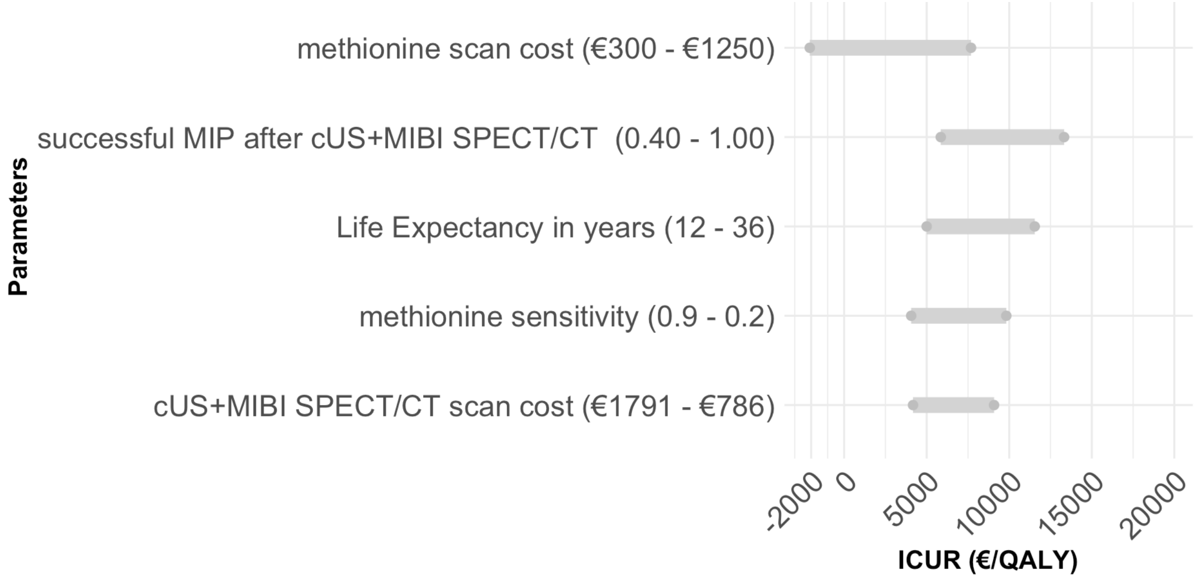

Figure 2: A tornado plot illustrating the impact of key parameters on the incremental cost-utility ratio (ICUR) of [^{11}C]methionine positron emission tomography/computed tomography. Methionine is compared to the standard approach of cervical ultrasound (cUS) combined with [^{99m}Tc]Tc-methoxy isobutyl isonitrile-single photon emission computed tomography/computed tomography (MIBI SPECT/CT). The modelled range for each parameter is indicated in brackets.
